# The systematic early integration of palliative care into multidisciplinary oncology care in the hospital setting (IPAC), a randomized controlled trial: the study protocol

**DOI:** 10.1186/s12913-015-1207-3

**Published:** 2015-12-15

**Authors:** Gaëlle Vanbutsele, Simon Van Belle, Martine De Laat, Veerle Surmont, Karen Geboes, Kim Eecloo, Koen Pardon, Luc Deliens

**Affiliations:** End-of-Life Care Research Group, Vrije Universiteit Brussel & Ghent University, Laarbeeklaan 103, 1090 Brussels, Belgium; Palliative care team, Ghent University Hospital, De Pintelaan 185, 9000 Ghent, Belgium; Department of Respiratory Medicine/Thoracic Oncology, Ghent University Hospital, De Pintelaan 185, Ghent, Belgium; Department of Gastroenterology, Division of Digestive Oncology, Ghent University Hospital, De Pintelaan 185, Ghent, Belgium; Department of Medical Oncology, Ghent University Hospital, De Pintelaan 185, 9000 Ghent, Belgium

**Keywords:** Palliative care, End-of-life care, Neoplasm, Advanced cancer, Quality of life, Multidisciplinary care, Mood, Informal caregiver, Study protocol

## Abstract

**Background:**

Previous studies in the US and Canada, have shown the positive impact of early palliative care programs for advanced cancer patients on quality of life (QoL) and even survival time. There has been a lack of similar research in Europe. In order to generalize the findings from the US and Canada research on a larger scale, similar studies are needed in different countries with different care settings. The aim of this paper is to describe the research protocol of a randomized controlled trial, situated in Flanders, Belgium, evaluating the effect of systematic early integration of palliative care in standard oncology care.

**Methods/Design:**

A randomized controlled trial will be conducted as follows: 182 patients with advanced cancer will be recruited from the departments of Medical Oncology, Digestive Oncology and Thoracic Oncology of the Ghent University Hospital. The trial will randomize patients to either systematic early integration of palliative care in standard oncology care or standard oncology care alone. Patients and informal caregivers will be asked to fill out questionnaires on QoL, mood, illness understanding and satisfaction with care at baseline, 12 weeks and every six weeks thereafter. Other outcome measures are end-of-life care decisions and overall survival time.

**Discussion:**

This trial will be the first randomized controlled trial in the Belgian health care setting to evaluate the effect of systematic early integration of palliative care for advanced cancer patients. The results will enable us to evaluate whether systematic early integration of palliative care has positive effects on QoL, mood and patient illness-understanding and which components of the intervention contribute to these effects.

**Trial registration:**

Clinicaltrials.gov Identifier: NCT01865396, registered 24^th^ of May, 2013.

**Electronic supplementary material:**

The online version of this article (doi:10.1186/s12913-015-1207-3) contains supplementary material, which is available to authorized users.

## Background

Patients with advanced and incurable cancer typically suffer from a multitude of severe symptoms, which often appear to be under-diagnosed. According to a European multicentre study, symptom intensity is underestimated in one in ten patients, with variations between cancer diagnoses [1]. This underestimation can be explained by a lack of expert knowledge in symptom management, limited time to tend to patients and a strong orientation among health care providers towards cure or life-prolongation rather than quality of life, leading to lack of therapeutic pertinacity [2]. There is evidence that all these problems can be tackled by early implementation of palliative care programs (PCPs) in standard oncological treatment [3–5]. Results from recent randomised controlled trials (RCTs) have demonstrated a positive impact on quality of life of PCP. One PCP for advanced cancer patients, situated in the US, consisted of a phone-based psycho-educational intervention led by a palliative care advanced practice-nurse. The RCT study of this intervention demonstrated positive effects on quality of life and to lower symptom intensity for cancer patients living in a rural setting [6]. Another RCT study, performed in Canada, implemented an intervention that consisted of a palliative care consultation by a palliative care physician and a palliative care nurse with advanced cancer patients with phone-based follow-up and a 24-h on-call service. Positive effects were reported four months after baseline for quality of life, symptom severity and satisfaction with care [7, 8]. Considered as a landmark study on integration of palliative care is the RCT study situated in a major university hospital in Boston, US [4, 9, 10] where the early palliative care intervention consisted of consultations with outpatients with metastatic non-small-cell lung cancer by a physician from the palliative care team shortly after diagnosis of advanced cancer and at least monthly thereafter. Patients in the palliative care group had significantly higher quality of life and a longer median survival of 2.8 months compared with those who received the standard care alone [4, 10, 11]. These promising results influenced the American Society of Clinical Oncology (ASCO) to support the full integration of palliative care as a routine part of comprehensive cancer care in the United States [12]. So far, all published results are from studies that have been conducted in the US and Canada; no results have been published on studies in Europe. Before it can become part of general clinical practice, this early palliative care approach has to be tested in different centres and most importantly in different countries where standard oncology care and/or palliative care may be different from that in the US or Canada.

In Belgium standard oncology care in the hospital is organised on a multidisciplinary model. At the medical level, specialists from different disciplines work closely with each other in diagnosing and treating the patient. At a paramedical level, many resources are devoted to ensuring psychosocial support for cancer patients; every hospital in Belgium with an oncology department has a psychosocial team available for patients and their family carers, consisting of a psychologist, a social worker, a dietician and a nurse specialist. Consultations with the professional caregivers of these teams are free of charge and a first consultation is often organized at the start of treatment. Because of government funding, all hospitals in Flanders, Belgium have a small palliative care team (palliative care nurse 0.5 FTE, palliative care physician 0.5 FTE and palliative care psychologist 0.5 FTE for every 500 hospital beds) available for patients who no longer receive curative treatment or care at the hospital. Palliative care teams are not structurally embedded into the psychosocial team for cancer patients but consultations with them are also free of charge. Patients are mostly seen by a palliative care nurse from the palliative care team [13]. Despite the availability and financial accessibility of palliative care teams, palliative care professionals are only involved in the care of about 60 % of patients with advanced cancer and they are often involved late in the disease trajectory of these patients [14], hence a programme which focuses on systematically providing palliative care early in the disease trajectory of advanced cancer patients is an innovative approach for cancer patients in Flanders, Belgium.

The systematic early integration of palliative care (IPaC) study is the first RCT study on early palliative care for advanced cancer patients to be conducted in the Belgian health care setting. The overall aim of the present paper is to describe the IPaC study protocol.

## Methods

### Study design

This is a phase III randomized controlled trial (RCT) in a hospital setting with advanced cancer patients receiving systematic early integration of palliative care with standard oncology care versus advanced cancer patients receiving standard oncology care alone. Study participants will be randomized in a 1:1 fashion to the intervention arm or standard care arm. Patients will be recruited from the Digestive Oncology, Thoracic Oncology and Medical Oncology departments of the Ghent University Hospital, the second largest hospital in Belgium. These departments have been selected for the trial because almost all cancer patients in the Ghent University Hospital are treated in these departments. Stratification will take place for the treating oncology department (Digestive Oncology, Thoracic Oncology and Medical Oncology) in order to prevent imbalances between groups in factors related to possible differences in standard care [15].

### Study participants

One hundred and eighty two patients with advanced cancer will be recruited by the treating oncologists. Table 1 describes the inclusion and exclusion criteria. Additionally, participants are given the possibility of identifying an important person in their life, either a relative or friend, to be involved in the study as an informal caregiver. The informal caregivers of patients who are randomised to the intervention group may also attend the consultations and hence frequently meet with the palliative care team as opposed to the informal caregivers of the participants in standard care. Table 2 describes the inclusion-criteria for the family carers.

**Table 1:** Inclusion and exclusion criteria for the patient

Inclusion-criteria	1. Patients with a solid tumour treated at the Medical Oncology department, Thoracic Oncology department or Digestive Oncology department of the Ghent University Hospital
	2. Patients within 12 weeks of a new diagnosis of illness progression or patients originating from another hospital who are within 12 weeks of receiving first-line treatment
	3. Patients with a life expectancy of approximately one year (assessed by the treating oncologist)
	4. Patients with an Eastern Cooperative Oncology Group (ECOG) performance status of 0, 1 or 2
	5. Patients with the ability to read and respond to questions in Dutch.
Exclusion-criteria	1. Patients under 18 years old
	2. Patients with impaired cognition
	3. Patients with more than one palliative care consultation since the onset of the disease
	4. Patients with one palliative care consultation in the six months before inclusion

**Table 2:** Inclusion criteria for the family carer

1. This person should either live with the patient or have in-person contact with him or her at least twice a week.	

### Data collection

Oncologists will describe the study to the eligible patients with the help of a data manager. The oncologists are asked to use the terms ‘palliative care’ and ‘palliative care team’ specifically while explaining the study, in order for the patient to be thoroughly informed and to avoid confusion when they receive the first visit from the palliative care team. The oncologists will obtain the patient’s written informed consent prior to enrolment. Our researchers will inform the informal caregiver of the patients and obtain written informed consent from them. Study participants will be randomized in a 1:1 fashion to the standard care arm or the intervention arm.

### The intervention

The intervention, primarily led by the palliative care nurse, will consist of four major components: the palliative care consultation, assessment, referring role and training.

#### Palliative care consultation

The intervention consists of systematic consultations with the palliative care nurses of the palliative care team of the Ghent University Hospital shortly after diagnosis of advanced cancer or of the progression of the disease. The palliative care team of the Ghent University Hospital consists of three palliative care nurses (2.5FTE), a palliative care psychologist (0.5FTE) and a palliative care physician (0.5FTE). This systematic early integration of palliative care will be in contrast to standard oncology care, where referral to palliative care is only at the request of physicians, nurses or patients and mostly occurs late in the disease trajectory. The participant receiving systematic early palliative care will meet with the palliative care nurses at least once a month and more if needed or requested by the patient. The palliative care physician will support and advise the palliative care nurses and will have at least one consultation every three months with the participant. All consultations will take place at a time when the patient is at the hospital for diagnostic or therapeutic reasons. The content of the palliative care consultations will be based on the holistic approach of palliative care and will focus on illness understanding and illness perception, symptom burden, psychological coping, spiritual coping and medical decision making by the patient and his or her informal caregiver.

#### Assessment

An interview form (see Additional file 1: Appendix 1) has been developed to aid in the structuring of each consultation and allows for an individual approach with the patient as well as the informal caregiver [4]. The aim of the members of the palliative care team is to comprehensively assess how the patient and informal caregiver present with regard to illness understanding and illness perception, symptom burden, psychological coping, spiritual coping and medical decision-making at every consultation. However, when a participant is not willing to discuss a specific topic, this will be respected. For symptom burden, assessment will not only be performed through personal inquiry based on the time allocation form but also through the use of the Edmonton Symptom Assessment Scale (ESAS) [16]. The different items of the ESAS will be filled out by the patient and will be discussed with the member of the palliative care team. The palliative care nurse or palliative care physician and the patient will systematically consider the previous item scores of the ESAS by using a graph that plots the evolution of the scores. This will provide an overview of the fluctuation of the symptoms over time [16].The palliative care team will report the amount of time spent on these different topics on the interview form.

#### Referring role

Because of their holistic approach to care of the patient, the members of the palliative care team will have an important role in reporting problems that arise during the consultation e.g. debilitating side effects of treatment to the oncology team and other health care professionals involved inside and outside the hospital. To encourage this referring role, the intervention will include regular communication between the members of the palliative care team and other professional caregivers 1) through weekly multidisciplinary meetings with the oncology team; 2) through weekly internal meetings with all the other members of the palliative care team and 3) through reporting in the electronic patient file and the palliative care team is required to complete a referral checklist at the end of every consultation [Additional file 2: Appendix 2] which includes an assessment of the need to contact other professional caregivers such as members of the oncology team, the psychologist, the general practitioner and the home palliative care services.

#### Training

Several information sessions will be organized for the members of the palliative care team on specific early cancer treatments and their possible side effects. The senior oncologists of each department will give a presentation with the focus on cancer etiology, cancer treatment (chemotherapy, radiotherapy, etc.), specific symptoms and side effects and an additional information session will be organized by the researchers on the implementation of the intervention, with topics such as the use and interpretation of the ESAS, the use of the interview form and the referral checklist.

#### Standard care

In addition to medical treatment, all patients with advanced cancer treated in the Ghent University Hospital receive extensive standard paramedical care. (1) A clinical oncology nurse specialist supports the patients throughout their disease trajectory by providing information on treatment and possible side effects and coordinates hospital appointments. This nurse is available during hospital visits and can be telephoned during working hours. (2) An oncology psychologist helps patients and informal caregivers to cope with the psychosocial implications of cancer and cancer treatment and provides further consultations for the patient and/or their family members at the request of the patient. (3) A dietician has a standard introductory consultation with cancer patients diagnosed with oesophageal cancer, stomach cancer, pancreatic cancer and head and neck cancers. All other cancer patients are screened for weight loss during their hospital stay or treatment and whenever significant weight loss is found, the dietician is consulted. (4) The social worker helps and supports patients and their relatives in dealing with the consequences of the disease and treatment in their everyday life. They make practical arrangements related to the organization of specific assistance at home and they provide information about financing, social security, insurance and transitions to residential care. The social worker attends at the request of the patient, the oncologist or the oncology nurse. (5) In routine clinical practice in the Ghent University Hospital, the members of the palliative care team are not structurally involved in the treatment trajectory of the cancer patient but at the request of the patient or at the discretion of the attending physicians, or a member of the paramedical team in cases of difficult symptom control and issues related to end-of-life care, such as advance directives. This referral mostly occurs late in the treatment trajectory.

### Outcome measures

#### Study objectives

The primary objective of this study is to assess the impact of this intervention’s systematic early integration of palliative care on the patient’s quality of life. The secondary objectives are (1) to assess the impact on the patient’s median survival time, mood and illness understanding, (2) the impact on informal caregivers’ quality of life, mood, illness understanding and satisfaction with care during treatment and after the death of the patient and (3) to examine whether systematic early integration of palliative care, alongside hospital treatment and treatment characteristics, influences advance care planning and end-of-life decision-making.

#### Measurement instruments

The socio-demographic and clinical characteristics of the patient will be collected at baseline by using questionnaires for the patient and the physician and by consulting the medical record.

##### Patient and informal caregiver questionnaires

Quality of life of the patient will be measured with the use of the European Organisation for Research and Treatment of Cancer Quality of Life Questionnaire (EORTC QLQ C30) and the McGill Quality of Life Questionnaire (MQOL) [17–19]. The MQOL has been selected because it addresses the existential aspects of quality of life as well as the physical, emotional and social aspects of quality of life. A recent review qualified both its content and construct validity as high [20]. The mood of the patient will be measured by two instruments: the Hospital Anxiety and Depression Scale (HADS) and the Patient Health Questionnaire 9 (PHQ-9) [21–24]. Illness-understanding will be measured by a forward-backward translation of the questionnaire developed by the researchers of the previously mentioned study of palliative care for patients with metastatic non-small-cell lung cancer [4]. Data on mood, quality of life, illness understanding and satisfaction with care of the informal caregivers will be obtained by self-assessment instruments: HADS, PHQ-9, the Short Form-36 Health Survey (SF-36) a questionnaire for illness understanding and by the FAMCARE respectively [25]. A forward-backward translated and tested version of the FAMCARE will evaluate the satisfaction with care of the informal caregivers [26].

The surveys filled out by patients and informal caregivers, their frequency and the timing of completion are detailed in Table 3. The patient will complete the baseline questionnaires before randomization and the follow-up questionnaires with regard to quality of life, mood and illness-understanding firstly after 12 weeks and thereafter every six weeks. Change from baseline to 12 weeks after inclusion is chosen because approximately 80 % of patients are expected to be alive at 12 weeks after inclusion [4]. The informal caregivers will complete the baseline questionnaire before randomization and the follow-up questionnaires firstly after 12 weeks and then every six weeks. If the patient dies the informal caregiver is asked to fill out the questionnaire once more 12 weeks after the death.

**Table 3:** Types of patient and informal caregiver questionnaires and frequency and timing of completion

	Week					
	Baseline	12	18	24	…	12 weeks after patient’s death
PATIENT FORMS						
EORTC QLQ C30 and MQOL	X	X	X	X	X	
PHQ-9 and HADS	X	X	X	X	X	
Illness-understanding Questionnaire	X	X	X	X	X	
INFORMAL CAREGIVERS FORMS						
General Health SF-36	X	X	X	X	X	X
PHQ-9 and HADS	X	X	X	X	X	X
FAMCARE	X	X	X	X	X	
Illness-understanding Questionnaire	X	X	X	X	X	

##### Oncologist questionnaires

In order to collect information on advance care planning and the making of end-of-life decisions (ELDs), the oncologist will be asked to fill out a validated Dutch questionnaire that has been used many times before in nationwide ELD-incidence studies in Flanders, Belgium. In accordance with previous studies, three main categories of ELDs will be considered: the withholding or withdrawal of potentially life-prolonging treatments (non-treatment decisions), the intensified alleviation of symptoms with a possible life-shortening effect and the administration or supply of drugs with the intention of shortening the patient’s life (physician-assisted death) [27, 28].

Data on outcomes related to treatment characteristics will be collected from the medical records of the patient, including survival time, intensity of chemotherapy, hospital admissions and frequency of contact with a psychologist/dietician/other health care professional. According to the definitions of Temel et al., patients will be classified as having had aggressive care if they meet one of the following criteria: 1) chemotherapy within 14 days of death, 2) no palliative care involvement in the last three months of life, or 3) admission to a palliative care unit three days or less before death [4].

#### Statistical analysis and sample size calculation

To compare baseline characteristics and study outcomes for patients between groups, Fisher’s exact tests, chi-square tests and the independent-samples t-test or the non-parametric equivalent Mann–Whitney *U* will be used. The effect of early palliative care on the primary outcome quality of life will be assessed by multivariate regression analyses adjusting for baseline scores. Survival analysis, i.e. Kaplan-Meier and a Cox proportional hazards model, will be used to examine the effect of systematic early integration of palliative care on the secondary outcome survival from the time of inclusion adjusting for demographic and clinical baseline characteristics. With regard to missing data, we will use the conservative approach of carrying the last observation forward for all missing data.

To detect a significant and meaningful difference in the change of the EORTC QLQ-C30 from baseline to 12 weeks, 118 patients are needed. With the proposed sample size of 59 for the two groups, the study will have a power of 80 % to yield a statistically significant result. This computation assumes a mean score difference of 12 and the common within-group standard deviation is 23.0. A difference of 10 points on the multi-items scales from 0 to 100 is considered meaningful (50;51). On the basis of the study on early palliative care in advanced lung cancer patients, it can be expected that approximately 80 % of the study participants will still be alive at 12 weeks after inclusion and drop-out for reasons other than death will amount to 15 % [4]. In other words, an overall drop-out rate of 35 % is to be expected, accounting for the planned inclusion of a total of 182 patients in the study.

#### Ethical considerations

Since the participants in the control group will receive extensive medical and paramedical care and will not be refused palliative care at their request, it is deemed that this research design is acceptable. The procedure of inclusion is organized in such manner that all patients will be informed firstly at inclusion by their treating oncologists and secondly by the data manager. The treating oncologist will spend time explaining the aim of palliative care in general and its specific aim within this study. They will provide an information sheet and an informed consent document to the patient. In a second phase, the data manager will have a consultation of a minimum of half an hour with the patient to explain the nature and procedure of the study in depth and to answer any questions participants might have. The participants will then be asked to give written informed consent and will be given a guarantee that: 1) the data will be treated confidentially, 2) the data will be stored anonymously for analysis later on, 3) study participation will be on a voluntary basis and withdrawal will be possible whenever the patient wishes and 4) non-participation or withdrawing from the study will have no influence on the future care of the patient. After providing informed consent, all patients will fill out the baseline questionnaires in the presence of the data manager so they will be able to request help if necessary. They will also be able to discontinue completing the questionnaire at any moment. Moreover, if they appear to be distressed as a result of filling out the questionnaire, psychologists from the oncology departments will be available to provide counselling.

#### Approval

Approval for this study was given by the Ethical Committee of the Ghent University Hospital, Flanders, Belgium.

## Discussion

The IPaC study is the first randomized controlled single centre phase III trial to implement an early palliative care approach in standard oncological care in the hospital setting in Belgium for patients with advanced cancer. Following a baseline assessment, patients and their informal caregivers will be randomized to either the intervention group where systematic early integration of palliative care will be implemented or to the control group where care will be provided as usual. Follow-up measurements allow the detection of differences in the quality of life, mood and illness understanding between patients in the intervention group and those in the control group.

Our systematic early integration of palliative care intervention is based on the original guidelines of the early palliative care intervention that was examined in the study for non-small lung cell cancer patients but adaptations have been incorporated to fit the specifics of the Belgian health care setting [4]. A first adaptation lies in the fact that the intervention is primarily led by the palliative care team nurses and not by the palliative care physician, due to the organizational structure of hospital-based palliative care in Flanders. The influence of a nurse-led intervention may appear to be less effective than a physician-led intervention but it may be less costly and financially more feasible [5]. A second adaptation is that the palliative care nurse leading the intervention also has a *referring role*. The medical and paramedical members of the oncology team may be able to fulfill the palliative care needs of the patient adequately but their goal often remains to prolong life. Palliative care embraces a different approach, focusing on the needs and expectations of the patient from a holistic point of view, which may lead to the eliciting of different information from the patient and the palliative care nurses who, in this trial, have an important role in referring this information to the oncology team. This interdisciplinary approach will broaden the view on care. A third adaptation consists of the semi-systematic format of consultations based on an interview form in which the important palliative care topics that have to be discussed and treated are listed. In addition, standard symptom assessment (ESAS) is incorporated to evaluate symptom intensity at each consultation and to provide an overview of the fluctuation of symptoms. Research has shown that most health care professionals agree that use of the ESAS enhances patient care because it helps patients in articulating their symptom issues and aids in following them up [29].

Several limitations exist with regards to the IPaC-study. It is a single-centre study in a tertiary hospital setting, which might limit the generalization of the results to other hospitals. On the other hand, the organization of the oncology departments and palliative care team are similar between hospitals in Belgium, having been developed on the basis of the same legislative initiatives. A second limitation is that participants and staff members are not blinded for allocation to the intervention group or control group. It is possible that the treating oncologists and other members of the oncology team are affected by the new approach and will implement the new insights with patients in the control group (crossover effect). However, the oncologists and other health care professionals of the oncology team will be well informed about the goals and design of the study and will be made aware of bias risks. A third limitation concerns the possibility of selection bias, since participants are asked for consent prior to enrollment. Participants more open to end-of-life discussions might be more prone to taking part in the trial than others.

This trial also has several strengths. Firstly, our study protocol consists of a detailed description of standard oncology care, which has a particular focus on providing psychosocial support for cancer patients and their caregivers. Such a description is needed since the organization of standard care has an important influence on this intervention, specifically on the referring role of the palliative care nurses. The inclusion of this explicit description demonstrates a different approach from other early palliative care RCTs. Extensive information has been provided regarding the functioning of the palliative care clinic and the roles of the palliative care clinician in these trials but little information is available concerning the organization of standard oncology care [4, 6–8, 30–32]. In the US, a broad range of supportive services are offered by a multidisciplinary cohort of oncology health professionals but the availability of these services varies greatly among institutions. Hence, extrapolation of this information to the organization of the standard care of the early palliative care trials situated in the US is not feasible [33]. Secondly, for our primary outcome measure we have selected a questionnaire (EORTC QLQ-C30) on quality of life which is used in clinical trials in Europe and has been studied intensively from the perspective of the clinical significance of scores [34].

One of the challenges of the trial is the recruitment of patients with advanced cancer early in the stage of the disease trajectory. A systematic review of early integration of palliative care in hospitals has shown that staff-specific barriers often occur due to the reluctance of physicians to communicate prognostic information to the patient and family and the perception by staff of PC as ‘terminal care’ [35]. Clinicians are often concerned that referral to PC will alarm patients and their informal caregivers [5]. Hence, introducing a clinical trial of PC to advanced cancer patients early in their disease trajectory may prove to be difficult. However, with the support of the head oncologist of each department and the disseminating of the results of previous research in early palliative care we expect that the trial will be successfully initiated in the different departments involved.

## Conclusion

The IPaC trial is a randomized controlled trial approved by the Ethical Committee that aims to assess whether the quality of life of patients with advanced cancer and their informal caregivers is enhanced by implementing systematic early integration of palliative care in a setting where multidisciplinary cancer care is part of standard oncology care. This trial will add to the evidence about the effectiveness of integrated palliative care programmes for cancer patients. We hope that this study will not only show whether systematic early integration of palliative care is effective for cancer patients in Belgium but will also provide an increased understanding of the contribution of the different components of the systematic early integration of palliative care intervention to their quality of life.

## Additional files

Additional file 1:
**Interview form.** (DOCX 12 kb) Additional file 2:
**Referral Sheet.** (DOCX 12 kb)

## Abbreviations

IPaC: Integration of Palliative Care
US: United Stated
QoL: Quality of Life
PCPs: Palliative Care Programs
RCT: Randomized Controlled Trial
ASCO: American Society of Clinical Oncology
FTE: Full Time Equivalent
ECOG: Eastern Cooperative Oncology Group
ESAS: Edmonton Symptom Assessment Scale
EORTC QLQ C30: European Organisation for Research and Treatment of Cancer Quality of Life Questionnaire
MQOL: McGill Quality of Life Questionnaire
HADS: Hospital Anxiety and Depression Scale
PHQ-9: Patient Health Questionnaire 9
SF-36: Short Form-36 Health Survey
ELDs: End-of-Life Decisions

## References

[1] Fisch MJ, Lee JW, Weiss M, Wagner LI, Chang VT, Cella D (2012). Prospective, observational study of pain and analgesic prescribing in medical oncology outpatients with breast, colorectal, lung, or prostate cancer. J Clin Oncol.

[2] Greaves J, Glare P, Kristjanson LJ, Stockler M, Tattersall MHN (2009). Undertreatment of nausea and other symptoms in hospitalized cancer patients. Support Care Cancer.

[3] Hearn J, Higginson IJ (1997). Outcome measures in palliative care for advanced cancer patients: a review. J Public Health Med.

[4] Temel JS, Greer JA, Muzikansky A, Gallagher ER, Admane S, Jackson VA (2010). Early palliative care for patients with metastatic non-small-cell lung cancer. N Engl J Med [Internet].

[5] Bauman JR, Temel JS (2014). The Integration of Early Palliative Care With Oncology Care: The Time Has Come for a New Tradition. J Natl Compr Cancer Netw.

[6] Bakitas M, Lyons K, Hegel M (2009). The Project ENABLE II Randomized Controlled Trial to Improve Palliative Care for Patients with Advanced Cancer. J Am Med Assoc.

[7] Zimmermann C, Swami N, Krzyzanowska M, Hannon B, Leighl N, Oza A (2014). Early palliative care for patients with advanced cancer: a cluster-randomised controlled trial. Lancet.

[8] Zimmermann C. Effectiveness of Specialized Palliative Care : A systematic review. J Am Med Assoc. 2014;299(14):1698-1709. 10.1001/jama.299.14.1698 18398082

[9] Temel JS, Greer J, Admane S, Gallagher ER, Jackson V, Lynch TJ, et al. Longitudinalperceptions of prognosis and goals of therapy in patients with metastatic non-small-cell lung cancer: Results of a randomized studyof early palliative care. J Clin Oncol. 2011;29(17):2319–26. 10.1200/JCO.2010.32.4459 21555700

[10] Pirl WF, Greer JA, Traeger L, Jackson V, Lennes IT, Gallagher ER (2012). Depression and survival in metastatic non-small-cell lung cancer: effects of early palliative care. J Clin Oncol [Internet].

[11] Greer JA, Pirl WF, Jackson VA, Muzikansky A, Lennes IT, Heist RS (2012). Effect of early palliative care on chemotherapy use and end-of-life care in patients with metastatic non-small-cell lung cancer. J Clin Oncol.

[12] Smith TJ, Temin S, Alesi ER, Abernethy AP, Balboni TA, Basch EM (2012). American Society of Clinical Oncology provisional clinical opinion: the integration of palliative care into standard oncology care. J Clin Oncol.

[13] Zorg FEP (2014). Evaluatierapport palliatieve zorg.

[14] Beernaert K, Cohen J, Deliens L, Devroey D, Vanthomme K, Pardon K (2013). Referral to palliative care in COPD and other chronic diseases: a population-based study. Respir Med Elsevier Ltd.

[15] Nichol AD, Bailey M, Cooper DJ (2010). Challenging issues in randomised controlled trials. Injury.

[16] Bruera E, Kuehn N, Miller MJ, Selmser P, Macmillan K (1991). The Edmonton Symptom Assessment System (ESAS): a simple method for the assessment of palliative care patients. J Palliat Care.

[17] Aaronson NK, Ahmedzai S, Bergman B, Bullinger M, Cull A, Duez NJ (1993). The European Organization for Research and Treatment of Cancer QLQ-C30: a quality-of-life instrument for use in international clinical trials in oncology. J Natl Cancer Inst.

[18] Cohen SR, Mount BM, Bruera E, Provost M, Rowe J, Tong K (1997). Validity of the McGill Quality of Life Questionnaire in the palliative care setting: a multi-centre Canadian study demonstrating the importance of the existential domain. Palliat Med.

[19] Robin Cohen S, Mount BM (2000). Living with cancer: “Good” days and “Bad” days - What produces them? Can the McGill Quality of Life Questionnaire distinguish between them?. Cancer.

[20] Albers G, Echteld MA, de Vet HCW, Onwuteaka-Philipsen BD, van der Linden MHM, Deliens L (2010). Evaluation of quality-of-life measures for use in palliative care: a systematic review. Palliat Med.

[21] Katon WJ, Simon G, Russo J, Von Korff M, Lin EHB, Ludman E (2004). Quality of depression care in a population-based sample of patients with diabetes and major depression. Med Care.

[22] Zigmond AS, Snaith RP (1983). The hospital anxiety and depression scale. Acta Psychiatr Scand.

[23] Spinhoven P, Ormel J, Sloekers PP, Kempen GI, Speckens AE, Van Hemert AM (1997). A validation study of the Hospital Anxiety and Depression Scale (HADS) in different groups of Dutch subjects. Psychol Med.

[24] Kroenke K, Spitzer RL, Williams JB (2001). The PHQ-9: validity of a brief depression severity measure. J Gen Intern Med.

[25] Brazier JE, Harper R, Jones NM, O’Cathain A, Thomas KJ, Usherwood T (1992). Validating the SF-36 health survey questionnaire: new outcome measure for primary care. BMJ.

[26] Kristjanson LJ (1993). Validity and reliability testing of the famcare scale: Measuring family satisfaction with advanced cancer care. Soc Sci Med.

[27] Chambaere K, Bilsen J, Cohen J, Onwuteaka-Philipsen BD, Mortier F, Deliens L (2010). Physician-assisted deaths under the euthanasia law in Belgium: a population-based survey. CMAJ.

[28] Chambaere K, Bilsen J, Cohen J, Onwuteaka-Philipsen BD, Mortier F, Deliens L (2011). Trends in medical end-of-life decision making in Flanders, Belgium 1998-2001-2007. Med Decis Making.

[29] Bainbridge D, Seow H, Sussman J, Pond G, Martelli-Reid L, Herbert C (2011). Multidisciplinary health care professionals’ perceptions of the use and utility of a symptom assessment system for oncology patients. J Oncol Pract.

[30] Yoong J, Park ER, Greer JA, Gallagher ER JVA, Pirl WF (2013). Early palliative care in advanced lung cancer: a qualitative study. JAMA Intern Med.

[31] Jacobsen J, Jackson V, Dahlin C, Greer J, Perez-Cruz P, Billings JA (2011). Components of early outpatient palliative care consultation in patients with metastatic nonsmall cell lung cancer. J Palliat Med.

[32] Back AL, Park ER, Greer JA, Jackson VA, Jacobsen JC, Gallagher ER (2014). Clinician Roles in Early Integrated Palliative Care for Patients with Advanced Cancer. J Palliat Med.

[33] Deshields T, Zebrack B, Kennedy V (2013). The state of psychosocial services in cancer care in the United States. Psychooncology.

[34] Fayers P, Bottomley A (2002). Quality of life research within the EORTC—the EORTC QLQ-C30. Eur J Cancer.

[35] Dalgaard KM, Bergenholtz H, Nielsen ME, Timm H. Early integration of palliative care in hospitals: A systematic review on methods, barriers, and outcome. Palliat Support Care. 2014;12:1–19. 10.1017/S1478951513001338 24621947

