# Supplementary material for: The systematic early integration of palliative care into multidisciplinary oncology care in the hospital setting (IPAC), a randomized controlled trial: the study protocol
Source: BMC Health Serv Res. 2015 Dec 15;15:554. doi: 10.1186/s12913-015-1207-3 (PMC4678668; doi:10.1186/s12913-015-1207-3)
Supplement: Additional file 1: — Interview form. (DOCX 12 kb) [file 12913_2015_1207_MOESM1_ESM.docx]

**Appendices**

**Appendix 1**

| **Interview form Date of consultation:_____________________________** | |
| --- | --- |
| **Different topics:** | **How much of the time have you discussed the topic?** |
| Illness understanding–Illness perception –  Undertaken actions for eventual discomfort**:**  **___________________________________________________________________________**  **___________________________________________________________________________**  **___________________________________________________________________________**  **___________________________________________________________________________** | **…….%** |
| Symptom management – (use of ESAS)  Undertaken actions for eventual discomfort:  **___________________________________________________________________________**  **___________________________________________________________________________**  **___________________________________________________________________________**  **___________________________________________________________________________** | ……% |
| Dealing with a life-threatening illness- psychological:  Undertaken actions for eventual discomfort **___________________________________________________________________________**  **___________________________________________________________________________**  **__________________________________________________________________________**  **__________________________________________________________________________** | **………%** |
| Dealing with a life-threatening illness – spiritual:  Undertaken actions for eventual discomfort  **___________________________________________________________________________**  **___________________________________________________________________________**  **___________________________________________________________________________**  **_______________________________________________________________________** | **……..%** |
| Support in eventual decision at the end-of-life:  Undertaken actions for eventual discomfort  **___________________________________________________________________________**  **___________________________________________________________________________**  **___________________________________________________________________________**  **___________________________________________________________________________** | **……..%** |
| Other topics  Undertaken actions for eventual discomfort:  **___________________________________________________________________________**  **___________________________________________________________________________**  **___________________________________________________________________________**  **___________________________________________________________________________** | **……..%** |
