# Supplementary material for: The systematic early integration of palliative care into multidisciplinary oncology care in the hospital setting (IPAC), a randomized controlled trial: the study protocol
Source: BMC Health Serv Res. 2015 Dec 15;15:554. doi: 10.1186/s12913-015-1207-3 (PMC4678668; doi:10.1186/s12913-015-1207-3)
Supplement: Additional file 2: — Referral Sheet. (DOCX 12 kb) [file 12913_2015_1207_MOESM2_ESM.docx]

**Appendix 2**

**Referral Sheet:**

**Date of consultation:_____________________________**

1. Has the patient got any unanswered question regarding his/her treatment and visits to the hospital?

🞎 **No**

🞎 **Yes →** Please contact the **reference nurse**: NAME and TELEPHONE NUMBER

1. Has the patient got any severe and untreated symptoms due to the treatment, or specific questions regarding the treatment and medication?

🞎 **No**

🞎 **Yes →** Please contact the **treating physician** or **head** of the department: NAME and TELEPHONE NUMBER

1. Has the patient or his/her informal caregiver got any needs regarding specific social matters?

🞎 **No**

🞎 **Yes →** Please contact the **social nurse/social worker**: NAME and TELEPHONE NUMBER

1. Has the patient got any needs regarding nutrition?

🞎 **No**

🞎 **Yes →** Please contact the **dietician**: NAME and TELEPHONE NUMBER

1. Has the patient or his/ehr family got any care specific needs regarding psychological matters?

🞎 **No**

🞎 **Yes →** Please contact the **psychologis**t: NAME and TELEPHONE NUMBER

1. Is there a need to contact any external professional caregivers?

🞎 **No**

🞎 **Yes →** the **family physician**

**→** the **home care nurse**

**→** the **palliative home care team**
